# Metachronous Rectum Metastases from Gastric Adenocarcinoma: A Case Report

**DOI:** 10.1155/2012/726841

**Published:** 2012-11-25

**Authors:** Deniz Tural, Fatih Selçukbiricik, Abdülkadir Erçalışkan, Berin İnanç, Feray Günver, Evin Büyükünal

**Affiliations:** ^1^Division of Medical Oncology, Department of Internal Medicine, Cerrahpaşa Medical Faculty, İstanbul University, 34098 İstanbul, Turkey; ^2^Department of Radiation Oncology, İstanbul Education and Research Hospital, İstanbul, Turkey; ^3^Department of Pathology, İstanbul Education and Research Hospital, İstanbul, Turkey

## Abstract

*Introduction*. Hepatic metastases of gastric adenocarcinomas are frequently observed due to the drainage into portal vein. Intestinal metastases disseminate from gastrocolic and mesenteric ligaments but they are seen very rarely and in most cases detected in postmortem studies. *Case Report*. A 74-year-old female patient with no known history of disease. Her complaints on application were epigastric pain, burning, and constipation. Gastroscopy showed a submucosal mass in the greater curvature of fundus and in colonoscopy, a mass with polypoid appearance that narrows the lumen at the rectum was detected. No far metastases or pathology were detected. Pathology report from gastric biopsy material demonstrated well-differentiated adenocarcinoma. Cytokeratin 7 (CK7) was found to be extensively strongly positive, Cytokeratin 20 (CK20) was negative in the immunohistochemical staining of the biopsy obtained from rectosigmoid area. *Conclusion*. Gastric cancer is among the frequent cancers today, most of which are adenocarcinomas. Although most of the metastases are observed in the liver, lungs, lymph nodes, and peritoneum, it should be remembered that intestinal metastases may be seen without the presence of any other metastatic focus. Our case is the first in literature reporting a rectum metastasis without any other organ metastasis.

## 1. Introduction

Hepatic metastases of gastric adenocarcinomas are frequently observed due to the drainage into portal vein. In addition to peritoneal metastases while it is an intraperitoneal organ, other frequent areas of dissemination include lungs due to lymphatic drainage for the tumors adjacent to esophagocardiac junction and intra-abdominal lymph nodes. Intestinal metastases disseminate from gastrocolic and mesenteric ligaments but they are seen very rarely and in most cases detected in postmortem studies [1].

In our case, a rectal metastasis of an intestinal-type gastric adenocarcinoma was detected without any hepatic or lymph node metastases, the first of such case in literature. We aimed in our paper to present this case of rectal metastasis from primary gastric adenocarcinoma without any other metastatic foci by assessment from clinical, radiological, pathological, and genetic perspectives in light of up-to-date literature evidence.

## 2. Material and Method

The patient, who had epigastric pain and constipation was examined with endoscopy and colonoscopy. In pathological evaluation there was carcinoma both in stomach and rectum. Primary diagnosis was based on two different primary tumors and metachronous metastases. Thorax and abdominal computerized tomography (CT) was performed for other organ metastases. Differential diagnosis was made by the evaluation of the morphology and immunohistochemical staining of the carcinoma in stomach and rectum.

## 3. Case

**Figure 1:**
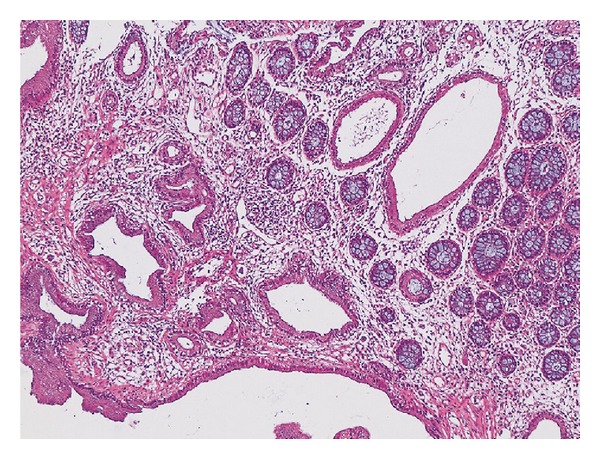
Microscopic image of the biopsy, stained with H&E, taken from fundus greater curvature by the use of gastroscopy. There is a well-differentiated adenocarcinoma infiltration forming irregular tubular structures in gastric mucosa (H&E, ×100).

A 74-year-old female patient with no known history of disease. Her complaints on application were epigastric pain, burning, and constipation. Gastroscopy showed a 2-cm submucosal mass in the greater curvature of fundus and in colonoscopy, a 2-3-cm mass with polypoid appearance that narrows the lumen at 10 cm in the rectum was detected. No far metastases or pathology were detected in the computer tomography of thorax/abdomen/pelvis, except thickening in the distal rectum wall and gastric wall. Biopsies were taken from the masses in the gastric and rectal areas. Pathology report from gastric biopsy material demonstrated well-differentiated adenocarcinoma (Figure 1).

**Figure 2:**
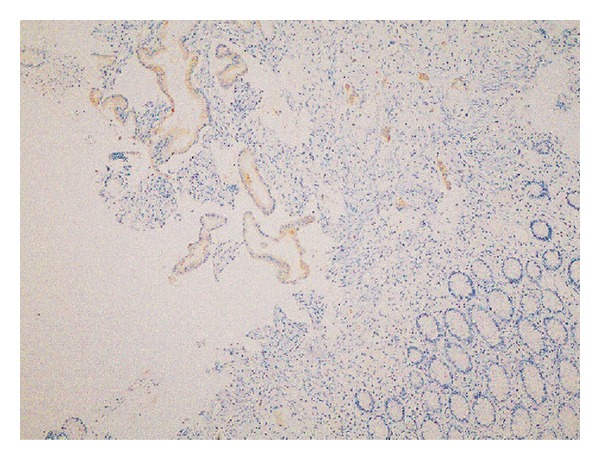
Immunohistochemical (IHC) staining of rectal biopsy taken during colonoscopy. Positive staining was detected in tumoral glandular structures by immunohistochemical examination of CK7. (CK7 is represented by light brown staining.) (IHC, CK7, ×100).

**Figure 3:**
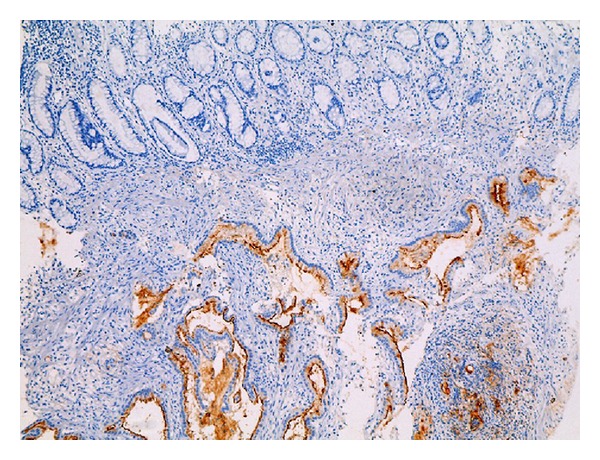
IHC staining of rectal biopsy taken during colonoscopy. While there was MUC1 positive staining in the tumor, there was no staining in rectum mucosal epithelium (MUC1 is represented by dark brown staining). (IHC, MUC1İ ×100).

**Figure 4:**
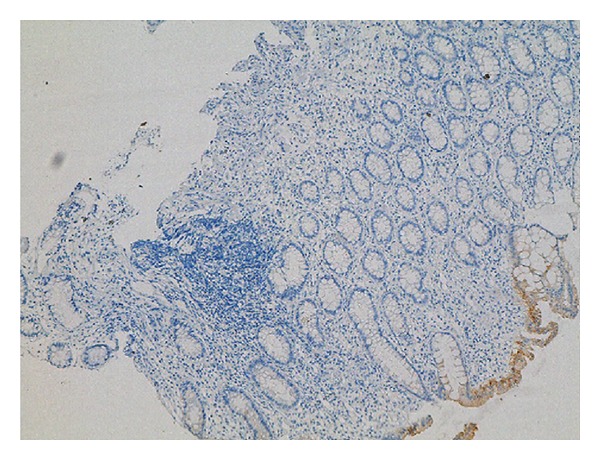
IHC staining of rectal biopsy taken during colonoscopy. While there was no staining of CK20 in the tumor, positive staining was detected in rectum mucosal epithelium. (CK20 is represented by dark brown staining) (IHC,CK20, ×100).

Cytokeratin 7 (CK7) (Figure 2) and musin 1 (MUC1) (Figure 3) were found to be extensively strongly positive, Cytokeratin 20 (CK20) was negative (Figure 4) in the immunohistochemical staining of the biopsy obtained from rectosigmoid area.

**Figure 5:**
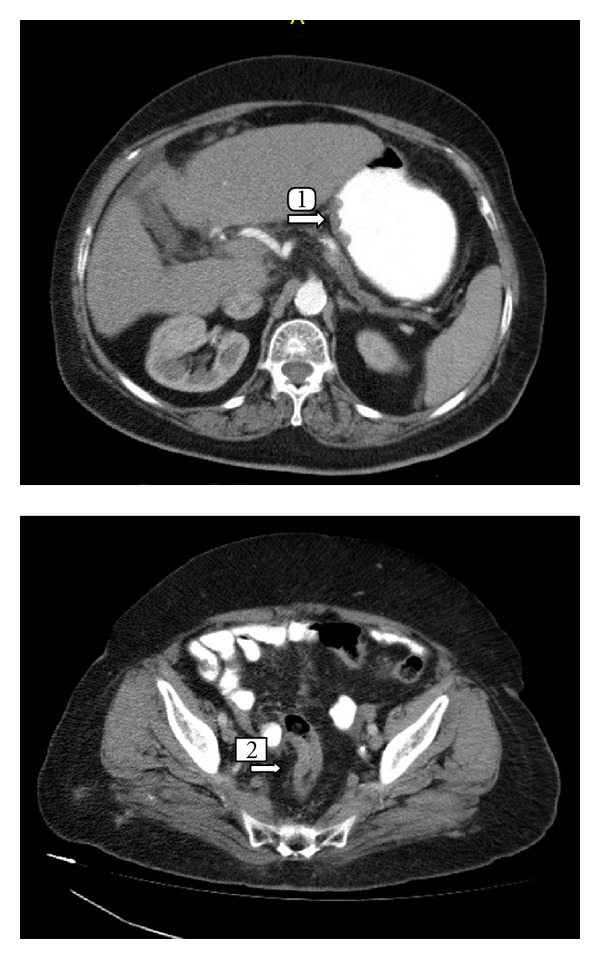
Evaluation of distant metastases by thorax and abdominal CT. No findings were detected in favor of additional metastatic foci in CT scans of thorax or abdomen other than thickening in the gastric wall and asymmetric thickening in the rectal wall. Arrow 1 indicates thickening in the gastric wall and arrow 2 indicates thickening in the rectal wall.

The rectosigmoid biopsy material was considered to be an infiltration from the gastric tumor, when assessed in light of morphologic findings and the results of immunohistochemical staining. No positive staining was detected in the cytoplasmic membrane in C-erb B2 immunohistochemical staining (Score 2). There was no amplification in C-erb B2 (Her 2-neu) gene with FISH method. No findings were detected in favor of additional metastatic foci in CT scans of thorax or abdomen other than thickening in the gastric wall and asymmetric thickening in the rectal wall (Figure 5).

Tumor markers CA 19-9 and CEA were found to be 3083 U/mg and 2 ng/dL, respectively. Chemotherapy was initiated at TCF protocol (cisplatin 75 mg/m^2^, docetaxel 75 mg/m^2^, and 5-fluorouracil 1000 mg/m^2^ from day 1 to 5; each cycle performed every 21 days), which is administered to patients with metastatic gastric cancer in accordance with NCCN 2012 guideline. Total neutrophil count was 398 following the 1st chemotherapy. This was considered as neutropenia grade 4 as per Common Terminology Criteria for Adverse Events (CTCAE) Version 4.0, and therapy was resumed decreasing the chemotherapy doses by 25%. No metastatic findings were present and there was regression in constipation in the interim assessment after 3 cycles, and CA 19-9 marker level was decreased to 380 ng/dL. Followup and treatment of the patient is still continuing.

## 4. Discussion

While the frequency of gastric cancers is gradually increased, the estimated incidence of gastric cancer in America was reported to be 21,260, and the number of deaths due to this cause was 11,210 in 2007 [2]. In 2000, it was estimated that there were 876,340 cases of gastric cancer worldwide, resulting in approximately 650,000 deaths [3]. Gastric malignancies are most frequently observed in the distal stomach (40%) and in particular in the antrum, followed by fundus and esophagogastric junction (35%) and corpus (25%) [4], for which adenocarcinomas again account for 90–95% [5].

Hepatic metastases of gastric adenocarcinomas are frequently observed due to the drainage into portal vein. In addition to peritoneal metastases while it is an intraperitoneal organ, other frequent areas of dissemination include lungs due to lymphatic drainage for the tumors adjacent to esophagocardiac junction and intra-abdominal lymph nodes. Intestinal metastases disseminate from gastrocolic and mesenteric ligaments but they are seen very rarely and in most cases detected in postmortem studies [1]. Rectal metastases of gastric adenocarcinomas are reported in the literature. In these cases, intestinal metastases of poorly differentiated diffuse signet ring cell-type gastric adenocarcinomas was identified and surgical therapy and/or chemotherapy was administered [6–8]. In our case, rectum metastasis of a moderately differentiated intestinal-type gastric adenocarcinoma without hepatic or lymph node metastases was detected.

Gastroscopy is the most effective method for establishing the diagnosis of gastric cancer. It is highly valuable in diagnosis because it allows direct visualization of the tumor, cytological tests and histological biopsy. In a study issued in 1998, sensitivity, specificity, positive predictive value, and negative prognostic value of gastroscopy was found to be 82%, 100%, 99.1%, and 99.6%, respectively. Taking into consideration all the data, it was found to be 99.6% diagnostic [9]. Endoscopic ultrasound is a valuable diagnostic tool in determining the invasion depth of tumor, despite being poorly successful in detecting regional lymph node metastases [10, 11]. In a study comparing imaging methods in detecting hepatic metastases of gastrointestinal cancers in 2002, sensitivity rates at equivalent specificity for MRI, CT, and US were found to be 76%, 72%, and 55%, respectively [12]. No findings were observed except a 1-cm cystic lesion and ascites in the abdominal US of our case. No findings in favor of metastasis were found in dynamic abdominal MRI and thoracic and abdominal CT scans. Immunohistochemical staining may be a lodestar in hepatic metastases with unknown primary focus. In a study issued in 1999, CK20 positivity was detected in 78% (94%, when adenocarcinoma metastases of gastric origin are excluded) of hepatic metastases from the malignancies originating from colon and rectum. Positive staining with CK20 was 65% extensive and 6% focal and CK7 staining was 6% positive in metastases with primary tumor in the liver. For those with primary tumor in the rectum, extensive CK20-positive staining was 83% where none was stained with CK7. Although positivity of CK20 and CK7 was detected in 74% (92%, when adenocarcinoma metastases of gastric origin are excluded) of hepatic metastases of the malignancies of pancreaticobiliary origin, variable staining patterns were seen in metastatic gastric adenocarcinomas. Gastric adenocarcinomas with hepatic metastases were stained 30% extensively and 20% focally with CK20, and 60% extensively with CK7, while 40% negatively with CK20 and CK7. Therefore, CK7 and CK20 stains do not seem to have a guidance attribution in determining the primary focus. In conclusion, a significant association was found with only CK20-positive staining between tumors originating from colon, rectum, pancreas, and biliary ducts with hepatic metastasis [13]. In the immunohistochemical staining of gastric biopsy preparations from our case, CK20 staining in tumor cells was negative and CK7 staining was extensively strongly positive. On the other hand, mucin-positive staining indicates poorer prognosis in gastric adenocarcinomas [14]. In a study published in 2001, mucin-positive adenocarcinoma cases, compared with mucin-negative ones, were seen in younger patients at lower localization in the stomach, with increased cancer depth, with larger tumor diameters and higher incidences of lymph node metastasis. Also lymphatic and venous permeation were more frequent in mucin-positive staining adenocarcinomas. Comparison of lymph node metastasis in MUC-negative and -positive staining carcinomas, as the cancer invaded the submucosa, the frequency of lymph node metastasis was increased in MUC-positive staining carcinomas. In our case, in spite of MUC1-positive staining and submucosal invasion, no lymph node metastasis was detected. Another important consideration in gastric adenocarcinomas is the amplification in C-erb B2 gene (HER2/neu gene, 17q12-q21.32). Proteins that form as a result of the amplification of this gene impact cell growth, migration, and differentiation by binding to HER2/neu receptors on the cellular membrane. Trastuzumab that binds these defective proteins is included in the management of gastric cancer today [15]. In a study published in 2002, overexpression of the protein C-erb B2 was detected rather in well- and moderately differentiated gastric adenocarcinomas and was not found in signet cell-type gastric cancers or linitis plastic [16]. In our case, C-erb B2 gene amplification was not observed in moderately differentiated gastric adenocarcinomas. Surgery is the only curative method in the treatment of localized gastric adenocarcinomas. However, chemotherapy + radiation therapy is the recognized method for advanced stage and metastatic gastric adenocarcinomas [17]. Cisplatin+5-FU ± Docetaxel (CF-DCF) combination is among the most favored chemotherapy modalities. In a study comparing these dual and triple regimens, addition of docetaxel to the dual CF chemotherapy is found to significantly increase the time to progression, survival, and response rates [18]. However, in an article issued in 2007, dual and triple regimens involving docetaxel are referred to produce neutropenia more frequently compared to other regimens [19]. Thus, neutropenia developed in our case following the second cure of triple DCF chemotherapy, and the treatment was resumed after decreasing the dose. In conclusion, gastric cancer is among the frequent cancers today, most of which are adenocarcinomas. Gastroscopy is the most valuable diagnostic method. Although most of the metastases are observed in the liver, lungs, lymph nodes, and peritoneum, it should be remembered that intestinal metastases may be seen without the presence of any other metastatic focus. In our case, a rectal metastasis of an intestinal-type gastric adenocarcinoma was detected without any hepatic or lymph node metastases, the first of such case in literature.

## References

[1] Feczko PJ, Collins DD, Mezwa DG (1993). Metastatic disease involving the gastrointestinal tract. *Radiologic Clinics of North America*.

[2] Jemal A, Siegel R, Ward E, Murray T, Xu J, Thun MJ (2007). Cancer statistics, 2007. *CA: A Cancer Journal for Clinicians*.

[3] World Health Organization http://www-dep.iarc.fr/.

[4] Hermann RE (1993). Newer concepts in the treatment of cancer of the stomach. *Surgery*.

[5] Abeloff MD, Armitage JO, Niederhuber JE, Kastan MB, McKenna WG (2008). *Abeloff's Clinical Oncology*.

[6] Pacea U, Continoa G, Chiappaa A (2009). Metachronous colon metastases from gastric adenocarcinoma: a case report. *Case Reports in Oncology*.

[7] Uzunoğlu S, Çiçin İ, Karagöl H, Tanrıverdi Ö, Gençhellaç H, Usta U (2008). Rektuma Linitis Plastika Şeklinde Metastaz Yapan ve Primer Rektum Kanseri Gibi Görünen Mide Adenokarsinomu: Olgu Sunumu. *Trakya Üniversitesi Tıp Fakültesi Dergisi*.

[8] Lim SW, Huh JW, Kim YJ, Kim HR (2011). Laparoscopic low anterior resection forhematogenous rectal metastasis from gastric adenocarcinoma: a case report. *World Journal of Surgical Oncology*.

[9] Hosokawa O, Tsuda S, Kidani E (1998). Diagnosis of gastric cancer up to three years after negative upper gastrointestinal endoscopy. *Endoscopy*.

[10] Willis S, Truong S, Gribnitz S, Fass J, Schumpelick V (2000). Endoscopic ultrasonography in the preoperative staging of gastric cancer: accuracy and impact on surgical therapy. *Surgical Endoscopy*.

[11] Wang JY, Hsieh JS, Huang YS, Huang CJ, Hou MF, Huang TJ (1998). Endoscopic ultrasonography for preoperative locoregional staging and assessment of resectability in gastric cancer. *Clinical Imaging*.

[12] Kinkel K, Lu Y, Both M, Warren RS, Thoeni RF (2002). Detection of hepatic metastases from cancers of the gastrointestinal tract by using noninvasive imaging methods (US, CT, MR imaging, PET): a meta-analysis. *Radiology*.

[13] Tot T (1999). Adenocarcinomas metastatic to the liver: the value of cytokeratins 20 and 7 in the search for unknown primary tumors. *Cancer*.

[14] Kawamura H, Kondo Y, Osawa S (2001). A clinicopathologic study of mucinous adenocarcinoma of the stomach. *Gastric Cancer*.

[15] Hudis CA (2007). Trastuzumab—mechanism of action and use in clinical practice. *The New England Journal of Medicine*.

[16] Takehana T, Kunitomo K, Kono K (2002). Status of c-*erb*B-2 in gastric adenocarcinoma: a comparative study of immunohistochemistry, fluorescence in situ hybridization and enzyme-linked immuno-sorbent assay. *International Journal of Cancer*.

[17] Köhne CH, Wils JA, Wilke HJ (2000). Developments in the treatment of gastric cancer in Europe. *Oncology*.

[18] van Cutsem E, Moiseyenko VM, Tjulandin S (2006). Phase III study of docetaxel and cisplatin plus fluorouracil compared with cisplatin and fluorouracil as first-line therapy for advanced gastric cancer: a report of the V25 study group. *Journal of Clinical Oncology*.

[19] Ilson DH (2007). Docetaxel, cisplatin, and fluorouracil in gastric cancer: does the punishment fit the crime?. *Journal of Clinical Oncology*.

